# Positive and linear association of hepatic steatosis index with female infertility in US women: results from the National Health and Nutrition Examination Survey 2013–2018

**DOI:** 10.3389/fpubh.2025.1617550

**Published:** 2025-06-26

**Authors:** Xinjie Wu, Lin Zhang, Weihua Wu

**Affiliations:** ^1^Department of Clinical Medicine, Jining Medical University, Jining, China; ^2^Center for Reproductive Medicine, Affiliated Hospital of Jining Medical University, Jining, China

**Keywords:** female infertility, hepatic steatosis index, reproductive health, steatosis, NHANES

## Abstract

**Background:**

Female infertility is a global reproductive health challenge. The hepatic steatosis index (HSI) is a simple and non-invasive screening tool for steatosis. We herein explore the association of HSI with female infertility through the National Health and Nutrition Examination Survey (NHANES) 2013–2018.

**Methods:**

This cross-sectional study included 2,133 reproductive-age women from 15 U.S. states, with data collected through standardized questionnaires, physical examinations, and laboratory tests across three survey cycles (2013–2018). HSI was assessed based on body mass index, ALT/AST, sex, and diabetes status. Female infertility status was ascertained through standardized questionnaire items reflecting clinical diagnostic criteria (≥1 year of unprotected intercourse without conception), though not verified by medical records or fertility testing. Multivariable logistic regression analysis was used to explore the association between HSI and female infertility and to calculate the odds ratio (OR) and 95% confidence interval (CI). Restricted cubic spline (RCS) and stratified analyses were further employed to examine potential nonlinear relationships and subgroup disparities. Explored the factors affecting HSI through multivariate analysis.

**Results:**

A total of 2,133 reproductive-age women were enrolled, of whom 271 had infertility. There was no significant trend in HSI levels across cycles. In the fully adjusted model, HSI showed positive cross-sectional associations with self-reported infertility status (OR 1.02, 95% CI 1.01–1.04, *p* = 0.005). Compared to Q1, HSI at Q4 was associated with a 72% increase in the odds of female infertility (*p* = 0.003). Restricted cubic spline (RCS) analysis indicated that this association was linear (p for nonlinear = 0.9698). Stratified analyses suggested that this association was more pronounced among those <35 years of age and those with <high school education. RCS analyses based on age subgroups similarly indicated that this association was significant and linear among participants <35 years.

**Conclusion:**

HSI was positively associated with the odds of female infertility and demonstrated a linear dose–response association. These findings suggest that HSI may be used as an potential marker to screen reproductive age women at high risk of infertility, although further research is needed to validate its predictive utility and clinical applicability.

## 1 Introduction

Female infertility is defined as the failure of a woman of childbearing age to achieve a successful pregnancy within 1 year of normal sexual intercourse and without using any contraception (1). A household survey data from 277 demographic and reproductive health surveys indicates that the global estimated prevalence of infertility among women of reproductive age is more than 12% (2). Recent findings from the Global Burden of Disease study reveal that in 2021, the global prevalence of female infertility was 101.1 million cases, with age-standardized prevalence and years lived with disability increasing by approximately 0.68 and 33.1%, respectively, over the past three decades (3, 4). In the United States, approximately 12.7% of reproductive-age women seek infertility treatment each year (5). Moreover, recent studies indicate that infertility rates in the U.S. have remained relatively stable over the past decade, though disparities persist across racial, socioeconomic, and educational groups, with higher rates observed among non-Hispanic Black women and those with lower educational attainment (6, 7). Accumulating evidence suggests that female infertility significantly affects mental and physical health and may be strongly associated with the development of various comorbidities such as psychological distress, cardiovascular disease (CVD), female breast and reproductive system cancers, and metabolic dysfunction (6, 8, 9). Female infertility is a persistent global reproductive health problem with significant social and health consequences, and maintaining optimal reproductive health is essential for individual health, economic development and overall human well-being (10, 11). Therefore, enhancing reproductive health by identifying modifiable risk factors for female infertility and prioritizing targeted interventions is of great public health value.

Hepatic steatosis is a pathologic condition characterized by abnormal increase and accumulation of fat content in hepatocytes under different etiologies (e.g., metabolic disorders and alcohol consumption) (12, 13). Microvesicular steatosis is implicated in acute mitochondrial dysfunction and hepatic failure (13). A recent nationally representative cross-sectional study estimated the prevalence of hepatic steatosis to be 27.3% among U.S. adults (14). Obesity is a major risk factor for hepatic steatosis, and the prevalence of hepatic steatosis is significantly increased in individuals with overweight or obesity (15, 16). In addition, hepatic steatosis has been shown to be the result of an imbalance in lipid metabolism, which is strongly associated with metabolic disorders and other cardiometabolic risk factors such as insulin resistance (17). Therefore, hepatic steatosis is recognized as a marker of metabolic dysfunction (18). The common background of hepatic steatosis and metabolic syndrome provides a pathophysiologic basis for female infertility. Insulin resistance may contribute to female infertility by affecting the hypothalamic–pituitary-ovarian axis, leading to reproductive hormone imbalances (19). In addition, insulin resistance may potentially increase the risk of infertility in women by initiating oxidative stress, disrupting energy metabolism, and affecting oocyte development, embryo quality, and endometrial tolerance (20). Interestingly, recent clinical studies have suggested that insulin resistance-related surrogate markers may be associated with the odds of female infertility (21). However, whether hepatic steatosis is associated with the occurrence of female infertility remains unexplored.

The hepatic steatosis index (HSI), an economical, noninvasive screening tool proposed by Lee et al. (22), has been widely used to assess the presence and severity of hepatic steatosis with good sensitivity and specificity (23, 24). This marker integrates important risk factors for hepatic steatosis including metabolic dysfunction, hepatic enzyme markers, and sex to reflect the presence of hepatic steatosis (22). A retrospective analysis demonstrated a significant correlation between HSI and non-enhanced CT-diagnosed hepatic steatosis in multivariate regression analysis (25). A cohort study demonstrated that elevated HSI in early pregnancy was associated with an increased risk of gestational diabetes in Chinese pregnant women and was mainly mediated by altered lipid metabolism (26). A case–control study showed that HSI was positively associated with levels of hyperandrogenemia markers, including free testosterone and free androgen index, in patients with polycystic ovary syndrome (PCOS) (27). However, there is still a paucity of clinical studies exploring whether HSI is significantly associated with the prevalence of female infertility. Addressing this research gap could help reveal the potential of HSI as a novel marker of female infertility and help identify those at high risk for infertility and optimize personalized prevention strategies.

Given the shared pathophysiological pathways between hepatic steatosis, metabolic dysfunction, and reproductive health, we hypothesized that HSI, a marker of hepatic steatosis, may be associated with female infertility. In this study, we employed data from the National Health and Nutrition Examination Survey (NHANES) to examine the association of HSI with female infertility and to reveal the disparities of this association across demographic characteristics such as age, providing a potential theoretical basis for future research.

## 2 Methods

### 2.1 Study design and population

NHANES is a major program of the National Center for Health Statistics (NCHS) designed to assess the health and nutritional status of the U.S. ambulatory population. Since 1999, NHANES has collected data from participants on a two-year cycle, with data types including standardized questionnaires, physical examinations, and laboratory tests. Therefore, NHANES is a series of ongoing, population-based, large-sample nationally representative cross-sectional surveys characterized by multistage probability sampling. NHANES data collection protocols undergo rigorous quality control through the National Center for Health Statistics, including standardized interviewer training, instrument calibration, and external validation of laboratory *methods against CDC benchmarks.* All NHANES survey protocols have been approved by the NCHS Ethics Review Board, and all participants have provided written informed consent.

**Figure 1:**
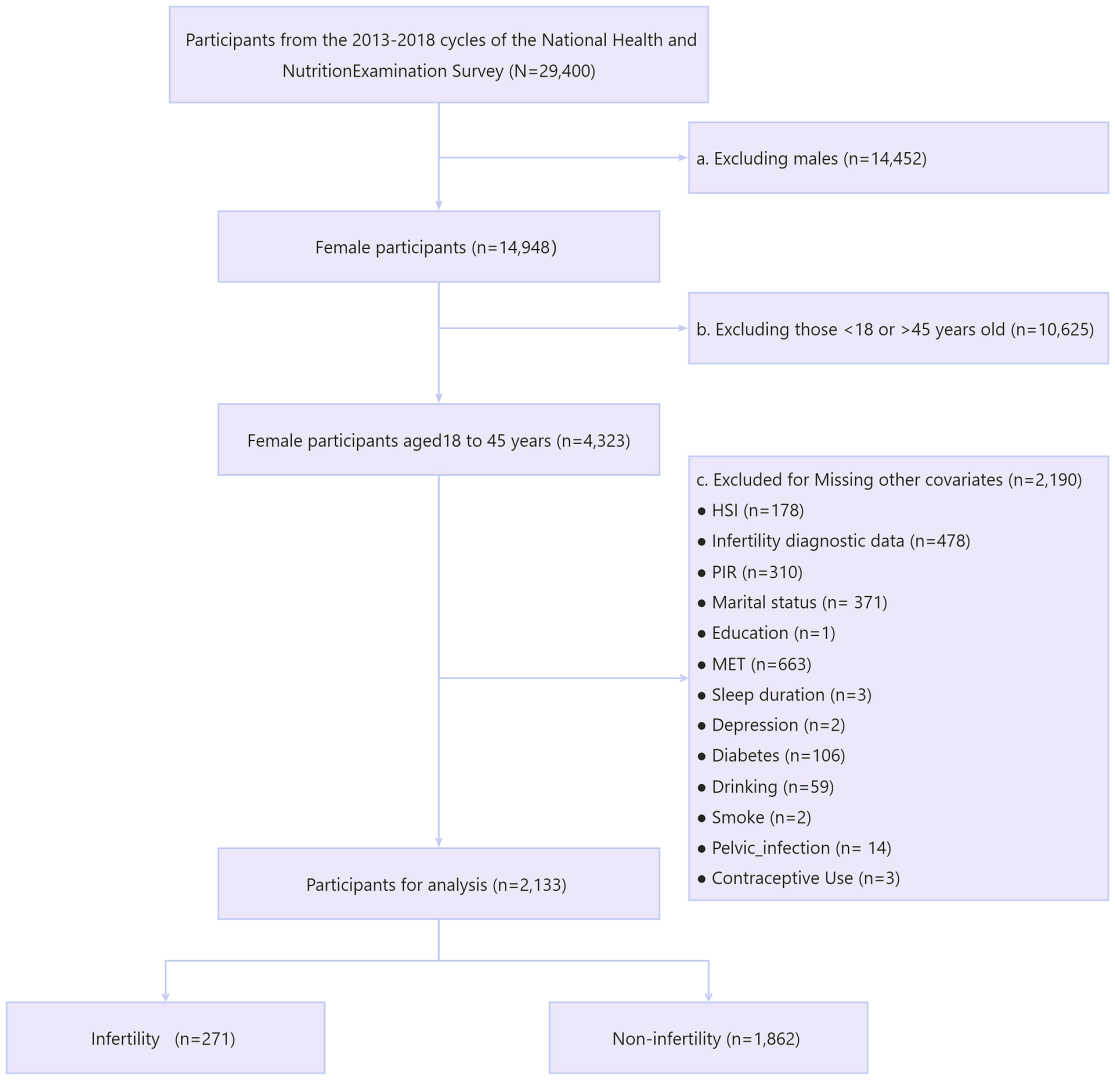
Flowchart of study population selection, NHANES 2013–2018.

We first included a total of 29,400 participants from three consecutive cycles of NHANES 2013–2018. Next, we sequentially excluded males (*n* = 14,452) and females not aged 18–45 (*n* = 10,625). We then excluded those with missing information on variables including HSI (*n* = 178), infertility (*n* = 478), income-poverty ratio (PIR) (*n* = 310), marital status (*n* = 371), education (*n* = 1), metabolic equivalents (MET) (*n* = 663), sleep duration (*n* = 3), depression (*n* = 2), diabetes (*n* = 106), alcohol consumption (*n* = 59), smoking (*n* = 2), pelvic infection (*n* = 14), and contraceptive use (*n* = 3). Ultimately, we included 2,133 eligible reproductive-age women for analysis, with 271 participants suffering from infertility (Figure 1).

### 2.2 Assessment of HSI

HSI is a non-invasive diagnostic tool used to assess the likelihood of fatty liver. The formula for calculating HSI is as follows: HSI = 8 × (ALT/AST) + BMI + 2 (plus 2 if female or diabetes) (22). ALT and AST were determined by laboratory tests on peripheral blood samples collected in the morning after fasting for at least 8 h. Serum ALT levels were measured using enzymatic assays, and AST levels were measured using kinetic rate assays. BMI was calculated by dividing weight (kg) by the square of height (m). Diabetes was assessed by self-reported history, abnormal laboratory tests (fasting blood glucose [FBG] ≥ 7.0 mmoL/L, 2-h postprandial blood glucose ≥11.1 mmoL/L, glycosylated hemoglobin A1c ≥ 6.5%], or taking antidiabetic medications (28).

### 2.3 Assessment of infertility

Female infertility was assessed through the reproductive health questionnaire in NHANES, which is designed to align with epidemiological definitions of infertility. Participants were interviewed on two related questions, “Have you ever attempted to become pregnant over a period of at least a year without becoming pregnant?” and “Have you ever been to a doctor or other medical provider because you have been unable to become pregnant?.” An affirmative answer to either of these two questions indicated the presence of female infertility, consistent with prior studies using NHANES data (7).

### 2.4 Covariates

We included a range of covariates including age, race (non-Hispanic White, non-Hispanic Black, Mexican American, or other race), education level (<high school, high school, or >high school education), PIR, marital status (married or non-married), smoking, alcohol consumption, sleep duration, physical activity, diabetes, hypertension, depression, pelvic infection, and contraceptive use. Smoking was assessed by smoking-related questionnaires and categorized as never smokers (<100 cigarettes in lifetime), former smokers (≥100 cigarettes in lifetime but now quit smoking completely), and current smokers (29). Participants’ drinking status was categorized through the Alcohol Use Questionnaire as follows: current drinkers: those who had at least 12 drinks in the past year; former drinkers: those who had consumed at least 12 drinks in their lifetime but fewer than 12 drinks in the previous year; and those who did not meet these criteria were categorized as never drinkers (30). Sleep duration was derived from participants’ self-reported average sleep time per night on the questionnaire. The MET was used to reflect the level of physical activity, which were calculated by the duration, frequency, and corresponding MET scores for each type of activity (31). Depression was assessed according to the Patient Health Questionnaire-9 (PHQ-9), where PHQ-9 ≥ 10 suggests the presence of major depression (32). Prediabetes was assessed according to impaired fasting glucose (FBG in the range of 5.6–6.9 mmoL/L) or impaired glucose tolerance (2-h oral glucose tolerance test 7.8–11.0 mmoL/L); diabetes was assessed by self-reported history, and abnormal laboratory tests, or taking antidiabetic medications as described above (28). Hypertension was assessed by a history of previous hypertension, blood pressure ≥140/90 mmHg, or taking hypertension medication (33). Pelvic infection and contraceptive use were assessed by participant self-report on the Reproductive Health Questionnaire.

### 2.5 Statistical analysis

Analyses incorporated NHANES examination weights, accounting for complex survey design through Taylor series linearization in the statistical analysis of this study to account for the complex survey design. We first analyzed the trends in HSI across successive cycles (2013–2014, 2015–2016, and 2017–2018) and explored the factors affecting HSI through multivariable linear regression analysis (34). Next, we performed baseline analyses based on HSI quartiles or infertility status. Continuous variables were expressed as mean ± standard deviation or median (quartiles) and were analyzed by weighted ANOVA, t-test, or nonparametric tests. Categorical variables were reported as numbers (percentages) and tested by weighted chi-square analysis. Multivariable logistic regression analyses were used to explore the association of HSI with the odds of infertility in reproductive-age women and to calculate odds ratios (ORs) and 95% confidence intervals (CIs). To avoid model multicollinearity, HSI as a continuous variable and HSI quartiles were analyzed in separate models. Crude models did not adjust for any covariates; model 1 partially adjusted for age and race; and model 2 additionally adjusted for education, marital status, PIR, depression, sleep duration, hypertension, diabetes, alcohol consumption, smoking status, MET, pelvic infection, and contraceptive use on top of model 1. Restricted cubic spline (RCS) modeling was used to explore whether there were nonlinear associations between HSI and infertility. In addition, we performed respective RCS analyses according to the age subgroups of participants (<35 and ≥35 years) to reveal whether this association differed by age. Stratified analyses were used to explore whether this association remained stable across the included subgroups of covariates, and interaction tests were used to analyze the factors that interacted with these associations. All statistical analyses were performed in R, with two-tailed *p* < 0.05 indicating statistical significance.

## 3 Results

### 3.1 Trends of HSI in different cycles and influencing factors

There was no significant change in the level of HSI across NHANES 2013–2014, 2015–2016, and 2017–2018 (*p* = 0.244) (Supplementary Figure S1). Consistently, when treated as a categorical variable, HSI quartiles did not change significantly across cycles (*p* = 0.52) (Supplementary Table S1). Multivariate analyses indicated that non-Hispanic White and other race (Mexican American as reference), PIR, sleep duration, hypertension, and prediabetes/diabetes (no diabetes as reference) influenced HSI levels (all *p* < 0.05) (Supplementary Table S2).

### 3.2 Baseline characteristics

Baseline analyses according to HSI quartiles indicated participants in higher HSI quartiles were older, had lower sleep duration, and were more likely to be Mexican American/non-Hispanic Black, ≤high school educated, have a PIR < 3.5, suffer from hypertension, prediabetes/diabetes, depression, and infertility (Table 1). Baseline analysis based on infertility status showed participants with infertility were older, slept fewer hours, and were more likely to be married, have hypertension, diabetes, depression, pelvic infection, and use contraceptives (Table 2).

**Table 1:** Baseline analysis according to HSI quartiles.

Variable	Total (*N* = 2,133)	Q1 (*N* = 533)	Q2 (*N* = 534)	Q3 (*N* = 533)	Q4 (*N* = 533)	*p* value
HSI	38.28 ± 0.31	28.42 ± 0.15	34.42 ± 0.08	40.86 ± 0.09	51.91 ± 0.29	<0.0001
Age, years	32.21 ± 0.25	30.48 ± 0.42	31.84 ± 0.44	33.86 ± 0.44	32.90 ± 0.38	<0.0001
Race						<0.0001
Mexican American	338(10.48)	40(4.93)	69(8.02)	122(15.33)	107(14.60)	
Non-Hispanic Black	444(12.10)	82(8.80)	90(9.49)	113(12.75)	159(18.11)	
Non-Hispanic White	790(60.77)	220(65.97)	220(66.97)	175(54.88)	175(53.80)	
Other	561(16.65)	191(20.30)	155(15.53)	123(17.03)	92(13.49)	
Education						<0.001
High school	378(17.16)	77(14.37)	81(13.92)	110(20.99)	110(20.09)	
Less than high school	259(8.37)	40(5.81)	60(6.82)	93(11.24)	66(10.10)	
More than high school	1,496(74.48)	416(79.82)	393(79.26)	330(67.77)	357(69.81)	
Marital status						0.120
Married	981(47.65)	215(42.73)	271(49.83)	248(48.91)	247(49.35)	
Non-Married	1,152(52.35)	318(57.27)	263(50.17)	285(51.09)	286(50.65)	
PIR						<0.001
<1.3	718(26.11)	146(21.58)	145(21.10)	212(30.10)	215(32.90)	
1.3–3.5	791(36.40)	173(31.36)	212(37.09)	197(37.41)	209(40.23)	
> = 3.5	624(37.48)	214(47.06)	177(41.81)	124(32.49)	109(26.88)	
Smoking						0.140
Never	1,501(67.61)	393(70.86)	400(70.66)	358(64.25)	350(63.89)	
Former	245(13.47)	57(13.30)	50(10.37)	70(15.47)	68(15.18)	
Now	387(18.92)	83(15.83)	84(18.97)	105(20.28)	115(20.93)	
Alcohol consumption						0.060
Former	113(4.46)	21(2.92)	25(4.85)	26(3.61)	41(6.58)	
No	321(11.42)	70(9.14)	96(13.09)	82(12.85)	73(10.58)	
Yes	1,699(84.12)	442(87.94)	413(82.06)	425(83.54)	419(82.84)	
Hypertension						<0.001
No	1761(84.91)	501(95.35)	474(90.66)	427(81.66)	359(69.96)	
Yes	372(15.09)	32(4.65)	60(9.34)	106(18.34)	174(30.04)	
Diabetes						<0.001
No	1870(89.31)	510(96.32)	511(96.66)	462(88.13)	387(74.25)	
Prediabetes	100(4.38)	13(2.48)	15(2.38)	32(5.81)	40(7.33)	
Diabetes	163(6.31)	10(1.20)	8(0.97)	39(6.05)	106(18.42)	
Depression						0.030
No	1926(90.01)	496(92.62)	489(91.00)	476(89.24)	465(86.75)	
Yes	207(9.99)	37(7.38)	45(9.00)	57(10.76)	68(13.25)	
Pelvic infection						0.160
No	2034(95.95)	518(97.49)	513(96.46)	507(95.56)	496(94.06)	
Yes	99(4.05)	15(2.51)	21(3.54)	26(4.44)	37(5.94)	
MET, minutes per week	4530.15 ± 151.72	4469.99 ± 305.26	4279.80 ± 291.58	4587.23 ± 306.55	4826.95 ± 327.47	0.650
Sleep duration, hours	7.47 ± 0.04	7.64 ± 0.07	7.48 ± 0.08	7.35 ± 0.07	7.41 ± 0.08	0.020
Contraceptive use						0.770
No	646(23.45)	170(24.75)	166(22.22)	165(24.20)	145(22.65)	
Yes	1,487(76.55)	363(75.25)	368(77.78)	368(75.80)	388(77.35)	
Infertility						<0.001
No	1862(86.25)	493(90.31)	480(89.17)	460(86.64)	429(77.96)	
Yes	271(13.75)	40(9.69)	54(10.83)	73(13.36)	104(22.04)	

Continuous variables: mean ± SD (weighted ANOVA/*t*-test). Categorical variables: n(%) (weighted chi-square test).

**Table 2:** Baseline analysis based on infertility status.

Variable	Total (*N* = 2,133)	Non-infertility (*n* = 1,862)	Infertility (*n* = 271)	*p* value
Age, years	32.21 ± 0.25	31.69 ± 0.24	35.47 ± 0.60	<0.001*
Race				0.160
Mexican American	338(10.48)	298(10.64)	40(9.48)	
Non-Hispanic Black	444(12.10)	385(12.24)	59(11.23)	
Non-Hispanic White	790(60.77)	676(59.86)	114(66.44)	
Other	561(16.65)	503(17.26)	58(12.85)	
Education				0.340
High school	378(17.16)	328(16.84)	50(19.15)	
Less than high school	259(8.37)	233(8.73)	26(6.07)	
More than high school	1,496(74.48)	1,301(74.43)	195(74.78)	
Marital status				<0.001*
Married	981(47.65)	810(44.61)	171(66.67)	
Non-Married	1,152(52.35)	1,052(55.39)	100(33.33)	
PIR				0.340
<1.3	718(26.11)	635(26.75)	83(22.15)	
1.3–3.5	791(36.40)	692(36.16)	99(37.94)	
> = 3.5	624(37.48)	535(37.09)	89(39.91)	
Smoking				0.270
Never	1,501(67.61)	1,328(68.42)	173(62.54)	
Former	245(13.47)	207(13.15)	38(15.47)	
Now	387(18.92)	327(18.43)	60(21.99)	
Alcohol consumption				0.100
Former	113(4.46)	95(4.05)	18(7.00)	
No	321(11.42)	291(11.88)	30(8.48)	
Yes	1,699(84.12)	1,476(84.06)	223(84.52)	
Hypertension				0.001*
No	1761(84.91)	1,559(86.34)	202(75.96)	
Yes	372(15.09)	303(13.66)	69(24.04)	
Diabetes				0.010*
No	1870(89.31)	1,644(89.97)	226(85.13)	
IGT	100(4.38)	86(4.39)	14(4.27)	
Yes	163(6.31)	132(5.63)	31(10.60)	
Depression				0.010*
No	1926(90.01)	1,695(90.86)	231(84.66)	
Yes	207(9.99)	167(9.14)	40(15.34)	
Pelvic infection				0.010*
No	2034(95.95)	1786(96.58)	248(92.00)	
Yes	99(4.05)	76(3.42)	23(8.00)	
MET, minutes per week	4530.15 ± 151.72	4485.08 ± 163.69	4812.74 ± 346.15	0.390
Sleep duration, hours	7.47 ± 0.04	7.51 ± 0.04	7.24 ± 0.10	0.010*
Contraceptive use				0.030*
No	646(23.45)	587(24.40)	59(17.46)	
Yes	1,487(76.55)	1,275(75.60)	212(82.54)	

^*^p < 0.05.

### 3.3 Association between HSI and female infertility

HSI was significantly associated with female infertility in the crude model and model 1 (crude model: OR 1.02, 95% CI 1.01–1.04; model 1: OR 1.03, 95% CI 1.01–1.04). After adjusting for all covariates, HSI remained positively associated with the odds of infertility (OR 1.02, 95% CI 1.01–1.04, *p* = 0.005). Compared to Q1, HSI at Q4 was associated with significantly increased odds of infertility (OR 1.72, 95% CI 1.23–2.40, *p* = 0.003). A significant trend was observed, with higher HSI quartiles associated with increased odds of infertility (p for trend = 0.01) (Table 3).

**Table 3:** Association of HSI with female infertility.

Character	Crude model		Model 1		Model 2	
	OR (95%CI)	P	aOR (95%CI)	P	aOR (95%CI)	P
HSI	1.02(1.01,1.04)	<0.001	1.03(1.01,1.04)	0.001	1.02(1.01,1.04)	0.005
HSI quartiles						
Q1	ref		ref		ref	
Q2	1.18(0.80,1.72)	0.39	1.12(0.77,1.63)	0.53	1.10(0.75,1.59)	0.610
Q3	1.25(0.85,1.83)	0.25	1.17(0.78,1.74)	0.44	1.13(0.74,1.73)	0.550
Q4	1.86(1.33,2.60)	<0.001	1.85(1.28,2.68)	0.002	1.72(1.23,2.40)	0.003
*p* for trend		0.001		0.004		0.010

OR: Odds ratio from crude model (unadjusted) and Model 1 (adjusted for age and race). aOR: Adjusted odds ratio from multivariable logistic regression (Model 2), adjusted for age, race, education, marital status, PIR, depression, sleep duration, hypertension, diabetes, alcohol consumption, smoking status, MET, pelvic infection, and contraceptive use. Continuous HSI and HSI quartiles were analyzed in independent models.

### 3.4 RCS analysis

**Figure 2:**
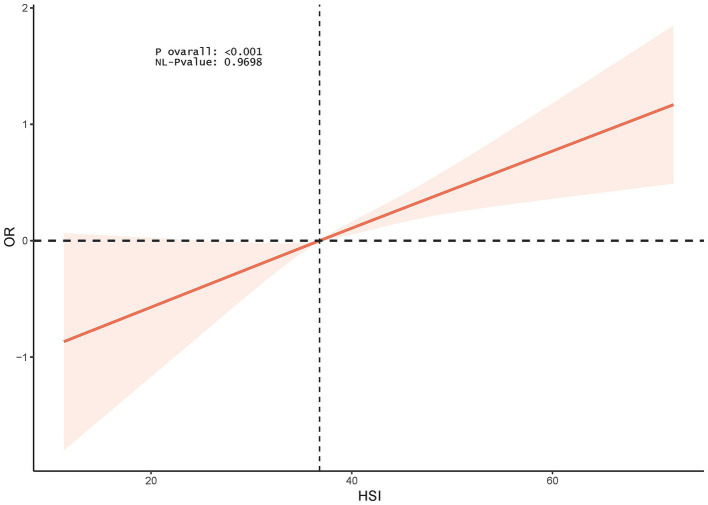
RCS analysis of the association between HSI and infertility.

The RCS model showed a linear association between HSI and female infertility (p for nonlinearity = 0.9698) (Figure 2).

### 3.5 Stratified analysis

**Figure 3:**
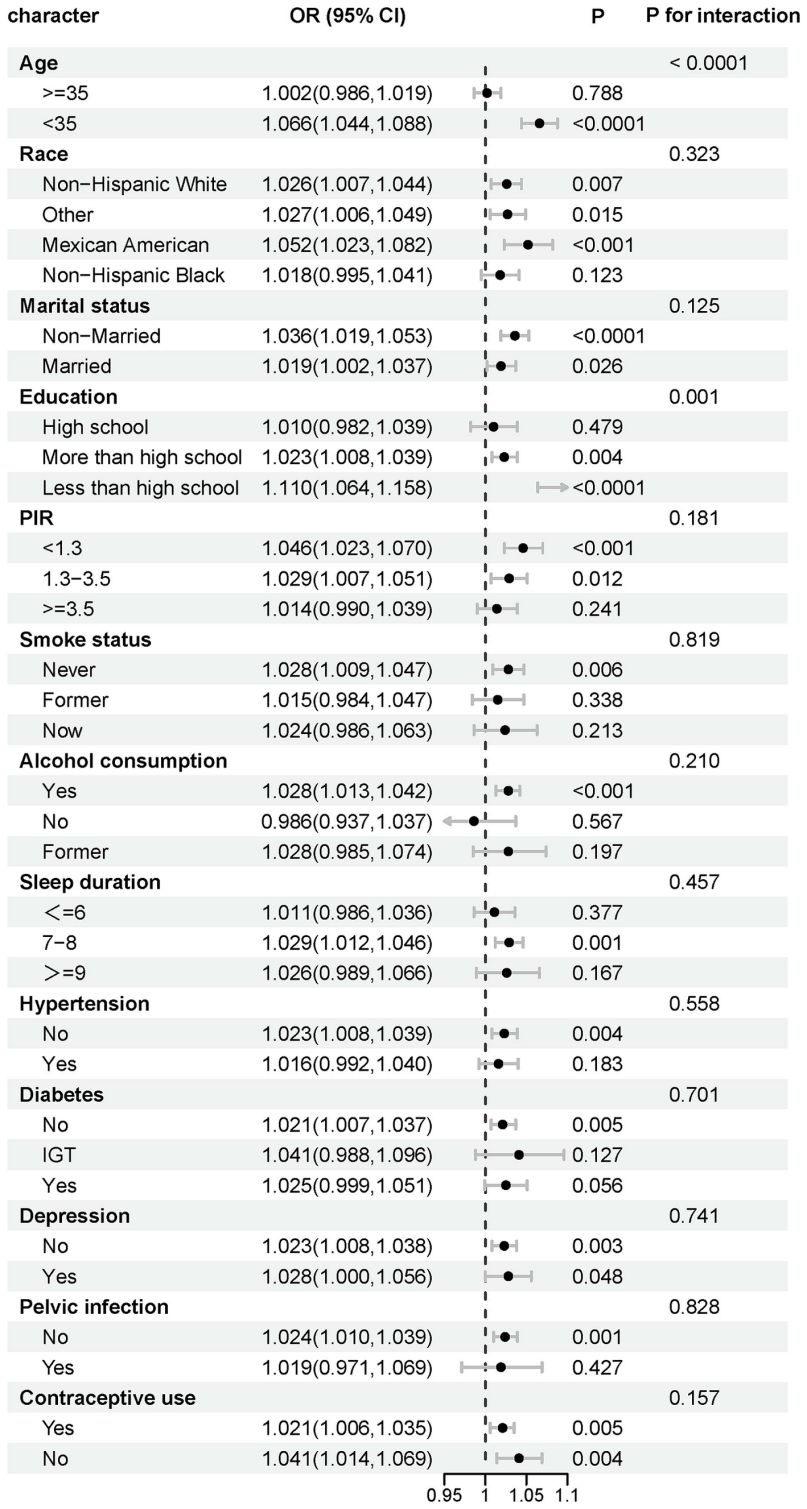
Stratified analysis of effect modifiers for the HSI-infertility association. Variables tested as potential effect modifiers via interaction terms.

**Figure 4:**
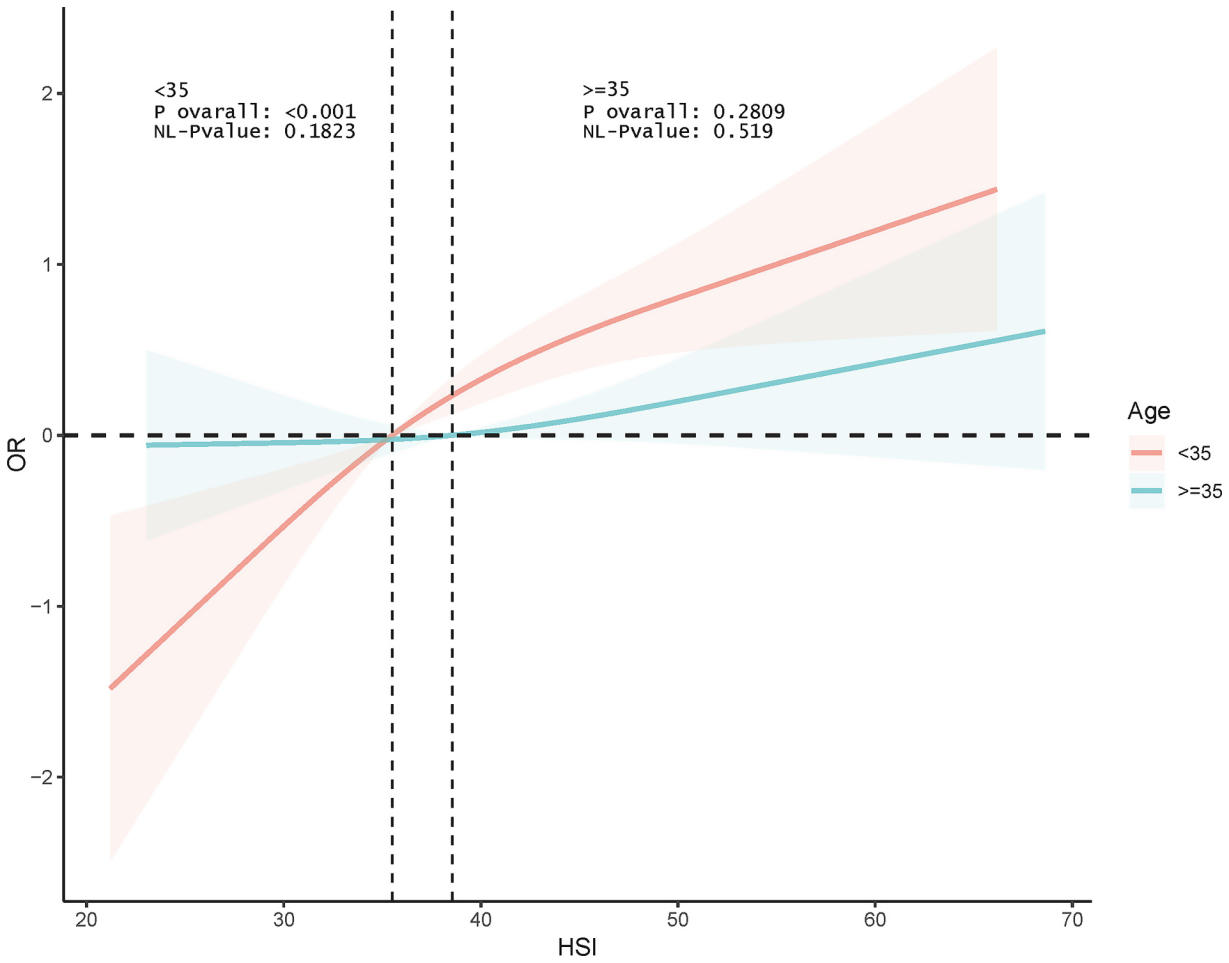
RCS analysis of the association between HSI and infertility according to age subgroups.

Interaction tests indicated that age and education were significant moderators of association (p for interaction <0.0001 and 0.001, respectively). This association was significant among participants <35 years old (OR 1.066, *p* < 0.0001) and disappeared among those ≥35 years old. In addition, this association was more pronounced among those with <high school education (OR 1.110, *p* < 0.0001) (Figure 3). RCS analyses according to age subgroups consistently showed this association to be significant and linear in those <35 years of age (p for overall <0.001, p for nonlinear = 0.1823) and non-significant at ≥35 years of age (p for overall = 0.2809) (Figure 4).

## 4 Discussion

In a national cross-sectional analysis, HSI was positively associated with the odds of infertility in reproductive-age women. After adjusting for all confounders, HSI at Q4 was associated with a 72% increase in the odds of infertility in women compared with Q1. The RCS model suggested that this association was linear. In addition, this association was more pronounced in people <35 years of age and <high school education. Suggest that HSI merits investigation as a potential risk indicator for early identification of people at high risk of infertility and individualized intervention to improve reproductive health.

To our knowledge, this is the first time that the association of HSI, a simple noninvasive marker of hepatic steatosis, with female infertility has been explored through a nationally representative study. Previous studies have demonstrated that HSI has an acceptable accuracy for the detection of hepatic steatosis and is in good agreement with imaging modalities. A cross-sectional study utilizing NHANES 2017–2018 demonstrated that HSI had the highest negative predictive value and sensitivity to exclude steatosis and the highest diagnostic odds ratio when using controlled attenuation transient elastography as a reference (35). A large body of evidence suggests that HSI can be used as a noninvasive biomarker for good differentiation of hepatic steatosis (23, 24, 36). A growing body of evidence suggests that HSI may be associated with outcomes in obstetrics and gynecology and have important clinical relevance. A prospective birth cohort study from China showed that HSI was significantly associated with an increased risk of gestational hypertension (OR 1.35, 1.86, and 3.81 for Q2, Q3, and Q4, respectively) and preeclampsia (OR 1.22, 1.96, and 3.60 for Q2, Q3, and Q4, respectively) (37). A recent cohort of pregnant women from China showed that HSI was significantly associated with a 1.67-fold (95% Cl: 1.05–2.67) increased risk of hypertensive disorders in pregnancy (38). A retrospective cohort study demonstrated that elevated HSI was associated with a composite of adverse maternal outcomes in adult women with singleton pregnancies who delivered at two tertiary hospitals (adjusted OR 1.55, 95% CI 1.11–2.17, *p* = 0.01) (39). An observational study from Italy showed that HSI was associated with impaired insulin sensitivity and compensatory increase in insulin release during pregnancy (40). Our study demonstrated for the first time that higher HSI was associated with increased odds of female infertility, independently of all confounding factors. These findings suggest that HSI may serve as an independent predictive marker of female infertility and emphasize the potential role of hepatic steatosis in the development of female infertility. While the per-unit increase in HSI showed a modest association with female infertility (adjusted OR = 1.02), the clinical significance is more meaningfully reflected in the quartile analysis. Women in the highest HSI quartile (Q4) exhibited a 72% increase in the odds of infertility compared to the lowest quartile (Q1). This magnitude of risk elevation is comparable to established risk factors for infertility such as polycystic ovary syndrome (PCOS) and obesity (47, 48). Such a substantial risk increase highlights the potential utility of HSI in identifying high-risk subgroups, particularly given that HSI integrates routinely measured clinical parameters (BMI, liver enzymes, diabetes status) into an easily calculable index. As a noninvasive and cost-effective tool, HSI may be used in large populations to screen individuals at high risk for female infertility and provide timely intervention to improve reproductive health. In addition,

Our findings also revealed that this association was more pronounced in those<35 years of age and<high school education. This finding supports focusing on the impact of elevated HSI on female infertility in these specific populations for individualized management and prevention.

The stronger association in women <35 years of age may reflect several factors: (1) Earlier metabolic dysfunction occurring during the prime reproductive years may have a more pronounced detrimental effect on ovarian function (19, 20); (2) Delayed infertility evaluation in older women (≥35 years) could lead to underreporting or misclassification of infertility causes, as these women may have already sought treatment or have age-related fertility decline overshadowing metabolic contributions (41); (3) Differential impact of insulin resistance on ovarian reserve, with younger ovaries being more sensitive to metabolic insults (21). Further studies are needed to elucidate the precise mechanisms underlying this age-specific effect.

Some studies suggest that HSI may be associated with insulin resistance and other cardiometabolic risk factors. One study showed that HSI was associated with insulin resistance and reduced levels of growth hormone and insulin-like growth factor 1 in patients with acromegaly (42). Another observational study including 92 non-diabetic patients demonstrated that HSI had the highest diagnostic value for hepatocellular fat content compared to other non-invasive markers (1H-magnetic resonance spectroscopy as reference) and was independently associated with insulin sensitivity and *β*-cell function (43). A cross-sectional study including 768 patients with type 2 diabetes mellitus showed that HSI was independently associated with carotid atherosclerosis (OR 1.174, 95% CI 1.174 1.123–1.228, *p* < 0.01) (44). A *post hoc* analysis of data from the Pathobiology of Prediabetes study in a bi-racial cohort showed that baseline HSI was associated with insulin sensitivity (*r* = −0.44, *p* < 0.0001), high-sensitivity C-reactive protein (*r* = 0.37, *p* < 0.0001) and adiponectin (*r* = −0.24, *p* < 0.0001) (45). At the subsequent 5-year follow-up, baseline HSI was significantly associated with risk of prediabetes (hazard ratio 1.138, 95% CI 1.027–1.261) (45). A population-based cross-sectional study demonstrated that triglycerides/high-density lipoprotein cholesterol (OR 6.55, 95% CI 1.17–36.46) and triglyceride-glucose index (OR 8.44, 95% CI 1.82–39.17, *p* < 0.05) were significantly associated with HSI in patients with PCOS (46). Collectively, these studies collectively suggest that HSI may be significantly associated with insulin resistance and other cardiometabolic risk factors such as systemic inflammation, thereby mediating the association with diseases.

The mechanisms underlying the association of HSI with female infertility remain unclear. We speculate that some possible biological mechanisms may explain this association. First, HSI has been shown to affect levels of hyperandrogenemia markers in patients with PCOS, potentially leading to reproductive hormone imbalances that promote the development of infertility (27). As mentioned, HSI is closely associated with insulin resistance. Recent studies have concluded that insulin resistance may affect the development of infertility in women through a variety of mechanisms, including effects on oxidative stress, energy metabolism, oocyte development, embryo quality, endometrial tolerance, hormone secretion, embryo implantation, and the effectiveness of assisted reproductive technology treatments in infertile populations (20). Further insights into the molecular mechanisms underlying HSI and female infertility are needed, especially the role of metabolic disorders, chronic inflammation and oxidative stress in this context.

There are several strengths to our study. Our study is based on the national representative, population-based, large-sample data from NHANES, making the results potentially replicable and generalizable. We adequately adjusted for confounding factors that could have influenced the association, reducing potential study bias. In addition, our study provides the first clinical investigation of the potential clinical value of HSI in the prediction and prevention of female infertility, which may have important clinical implications. However, our study has some important limitations. As a cross-sectional study, we were unable to determine the temporal order of the association, precluding the establishment of causality. Beyond the cross-sectional design and reliance on self-reported infertility data, our study has additional limitations. First, HSI, while a validated marker for hepatic steatosis, may not fully capture the severity or progression of liver disease, which could influence its association with infertility. Second, we did not account for potential confounders such as genetic factors or specific lifestyle behaviors (e.g., dietary patterns) that may mediate this relationship. Third, the study population was limited to U.S. women, and findings may not generalize to other populations with different demographic or health profiles. The diagnosis of female infertility was derived from participants’ self-reports and may have been influenced by recall bias, potentially leading to misclassification. The diagnosis of female infertility was derived from participants’ self-reports and may have been influenced by recall bias. However, the NHANES questionnaire interviews are conducted by trained personnel in a standardized manner and all self-reported diagnoses are ascertained by a physician or other professional, ensuring agreement with the true diagnosis. Finally, future studies need to further explore whether HSI can be used as a predictive marker of female infertility and evaluate its potential for application in clinical practice.

## 5 Conclusion

In a national cross-sectional analysis, HSI was positively associated with the odds of infertility in reproductive-age women and demonstrated a linear dose–response pattern. This management was more pronounced in those <35 years of age and <high school education. These findings suggest that HSI, as an economical and noninvasive tool, may serve as an emerging marker to help identify women at high risk for infertility However, further research is needed to validate its predictive utility and clinical applicability in optimizing personalized management strategies to prevent infertility development and improve the success of assisted reproductive technology.

## Data Availability

Publicly available datasets were analyzed in this study. This data can be found here: this study analyzed publicly available datasets and can be found at https://www.cdc.gov/nchs/nhanes/.
