# Supplementary material for: Positive and linear association of hepatic steatosis index with female infertility in US women: results from the National Health and Nutrition Examination Survey 2013–2018
Source: Front Public Health. 2025 Jun 26;13:1617550. doi: 10.3389/fpubh.2025.1617550 (PMC12241013; doi:10.3389/fpubh.2025.1617550)
Supplement: Supplementary file 1 [file Table_1.docx]

**Table S1**. Trends in levels of HSI quartiles across cycles.

|  | **Total** | **2013-2014** | **2015-2016** | **2017-2018** | **P value** |
| --- | --- | --- | --- | --- | --- |
| **HSI** |  |  |  |  | 0.52 |
| Q1 | 27.35(0.02) | 27.82(1.64) | 26.17(2.24) | 28.16(3.00) |  |
| Q2 | 26.29(0.02) | 27.20(1.21) | 26.30(1.57) | 25.30(2.34) |  |
| Q3 | 23.59(0.01) | 23.93(1.42) | 25.53(1.63) | 21.06(1.23) |  |
| Q4 | 22.77(0.01) | 21.05(1.77) | 22.00(2.11) | 25.48(2.83) |  |

**Table S2**. Multivariate analysis of factors affecting HSI levels.

| **character** | **Estimate** | **95% CI** | **Pr(>\|t\|)** |
| --- | --- | --- | --- |
| age | 0.05 | (-0.02, 0.13) | 0.18 |
| race |  |  |  |
| Mexican American | ref |  | ref |
| Non-Hispanic Black | -1.57 | (-3.31, 0.17) | 0.08 |
| Non-Hispanic White | -3.32 | (-5.02,-1.62) | <0.001 |
| Other | -3.9 | (-5.33,-2.47) | <0.0001 |
| Education |  |  |  |
| High school | ref |  | ref |
| Less than high school | -1.92 | (-4.07, 0.23) | 0.08 |
| More than high school | -0.73 | (-1.93, 0.48) | 0.23 |
| Marital status |  |  |  |
| Married | ref |  | ref |
| Non-Married | -1.03 | (-2.06,-0.01) | 0.05 |
| PIR | -0.77 | (-1.16,-0.37) | <0.001 |
| PHQ9 |  |  |  |
| no | ref |  | ref |
| yes | 1.12 | (-0.40, 2.64) | 0.14 |
| Sleep duration | -0.43 | (-0.80,-0.07) | 0.02 |
| Hypertension |  |  |  |
| no | ref |  | ref |
| yes | 4.72 | (3.16, 6.28) | <0.0001 |
| DM |  |  |  |
| no | ref |  | ref |
| predaibetes | 5.33 | (2.56, 8.10) | <0.001 |
| DM | 10.05 | (7.90,12.20) | <0.0001 |
| Drinking |  |  |  |
| former | ref |  | ref |
| no | -0.76 | (-3.05, 1.52) | 0.50 |
| yes | -0.33 | (-2.56, 1.89) | 0.76 |
| Smoking |  |  |  |
| never | ref |  | ref |
| former | 1.12 | (-0.74, 2.97) | 0.23 |
| now | -0.08 | (-1.28, 1.12) | 0.89 |
| Pelvic infection |  |  |  |
| No | ref |  | ref |
| Yes | 1.36 | (-1.31, 4.03) | 0.30 |
| Contraceptive use |  |  |  |
| No | ref |  | ref |
| Yes | 0.73 | (-0.53, 2.00) | 0.24 |
