# Supplementary figures and images for: Positive and linear association of hepatic steatosis index with female infertility in US women: results from the National Health and Nutrition Examination Survey 2013–2018
Source: Front Public Health. 2025 Jun 26;13:1617550. doi: 10.3389/fpubh.2025.1617550 (PMC12241013; doi:10.3389/fpubh.2025.1617550)

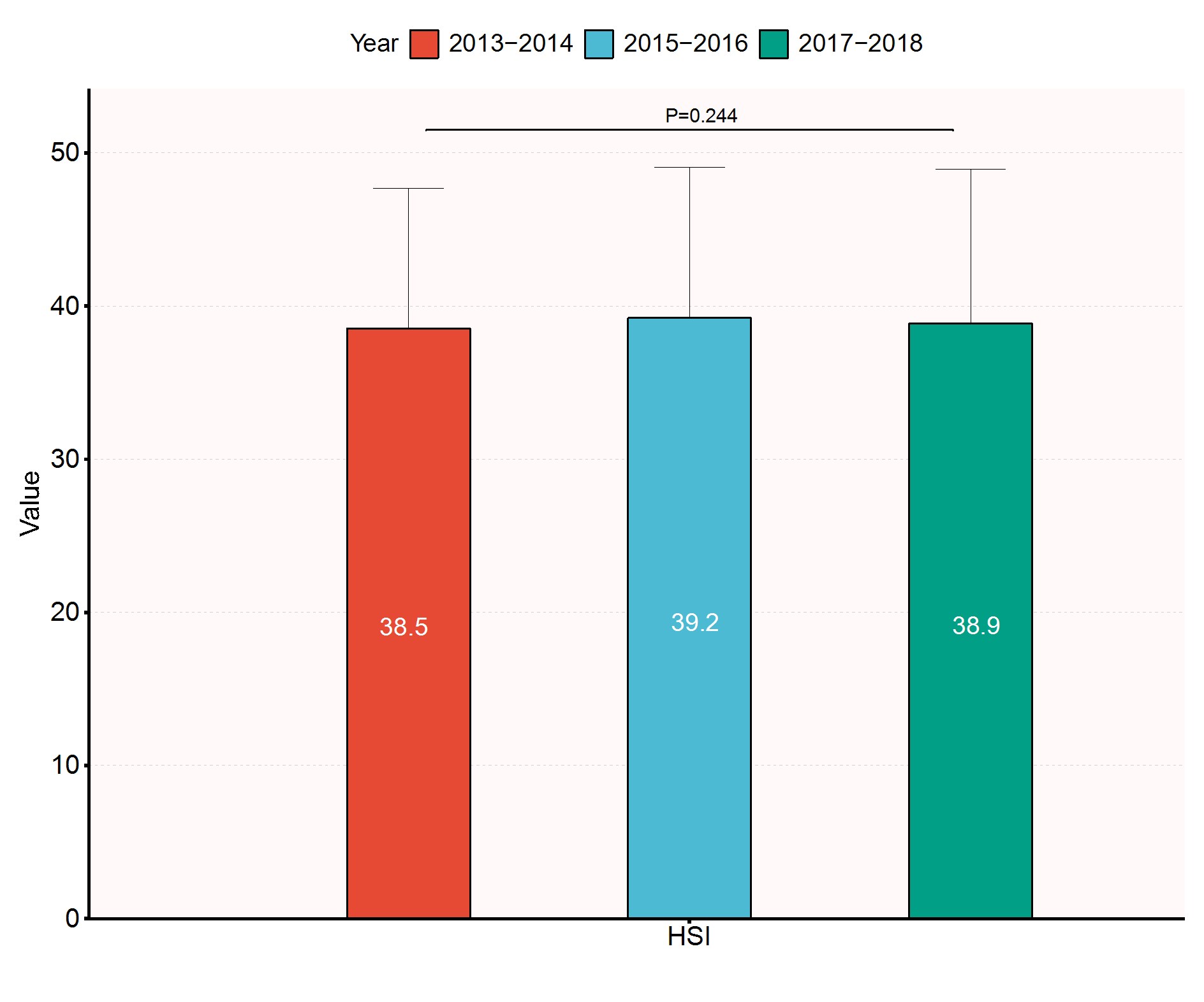

Supplement: Supplementary file 2 [file Image_1.jpeg]
